# The Critical Role of Race-Conscious Framework in Advancing Mental Health Workforce Diversity: A Case Study

**DOI:** 10.1007/s12552-026-09501-4

**Published:** 2026-03-07

**Authors:** Eric Kyere, Zohra Asad, Sadaaki Fukui

**Affiliations:** 1 Indiana University School of Social Work, Adjunct Associate Professor of Africana Studies, Indiana University, Indianapolis 902 West New York Street, Indianapolis, IN 46202-5156 USA; 2 Indiana University School of Social Work, 902 West New York Street, Indianapolis, IN 46202-5156 USA

**Keywords:** Workforce diversity, Mental health disparities, Racialized organization, Structural racism, Racial task burden, Racial outsourcing

## Abstract

Background: A racially diverse mental health workforce has been suggested to address persistent racial disparities in mental health among racially minoritized service recipients. However, in a racialized society such as the United States, structural racism is shown to constrain mental health organizations’ efforts to address disparities through workforce diversity. Theoretical Framework and Method: We recruited Black mental health workers (*n* = 10, M_age =_ 52.7 [SD = 6.9], 2 males (20%)/8 females; 4 married (40%)/6 single; 2 part time (20%)/8 full time) who have worked in a community mental health organization for at least seven years. We conducted semi-structured Zoom interviews with the participants to understand Black employees’ perceptions about the organization’s diversity efforts. Interviews were recorded, transcribed, and analyzed through the lens of the theory of racialized organizations, using the Sort and Sift, Think and Shift (SSTS) approach to qualitative data. Results: Findings were organized around five themes: (1) workforce diversity matters, (2) whiteness of the leadership as the perceptions of organizational diversity, and (3) the impact of the whiteness of leadership. Two related subthemes were identified from the third theme: (3a) racial task burdens and (3b) racial outsourcing. Discussion/Implications: Workforce diversity among racialized workers without focusing on how structural racism shape organizational processes are more likely to burden and exploit racial minority workers instead of promoting equity. Anti-racist work must move beyond a focus on individuals, as racist or bad actors, to target organizational procedures, operations, and resource allocation, which may have far greater consequences.

## Introduction

Organizational processes that promote the recruitment and retention of a racially diverse mental health workforce have been noted as an important pathway to address the persistent racial inequities linked to racial disparities in mental health in the United States (Damian et al., 2021; Kim, 2022; McGuire & Miranda, 2008). Such organizational processes enhance the effectiveness of healthcare workers with diverse backgrounds in serving recipients who are also racialized and marginalized individuals in communities with limited resources and healthcare facilities (Glazer et al., 2018; Rivera-Núñez et al., 2022). In this way, healthcare organizations with racially diverse workforce within a racialized society like the United States (US) foster racial concordance between healthcare service recipients from racialized groups toward improved quality of care (Glazer et al., 2018; Rivera-Núñez et al., 2022). Moreover, improved team functioning and reduced biases and mistrust have also been associated with a racially diverse healthcare workforce (White et al., 2021). Although ethical and relational sensitivity is needed to prevent potential intergroup conflicts among a racially diverse workforce (Maume et al., 2014), generally, there is a growing consensus that healthcare workforce, including mental health, with racial diversity holds some favorable effects for reducing disparities in mental health service access, usage, and outcomes among racially diverse client populations (Glazer et al., 2018; Rivera-Núñez et al., 2022).

However, given that the US is racially stratified, the healthcare system, including mental health organizations and their practices are shaped by structural racism both in historical and contemporary times (Shim & Vinson, 2020; Braveman et al., 2022; Castillo et al., 2020). Structural racism in organizations, including mental health, entails societal structures, cultural, and organizational ideas, rules, and practices that are grounded in, and operate interconnectedly to uphold, White logic, and whiteness in policies and practices (Bailey et al., 2021; Feagin, 2020; Gee & Hicken, 2021). That is, White logics and hegemonic whiteness— ideological and structural forces that legitimate norms, values, and culture that characterize the mainstream—structure organizational processes regardless of the existing demographic composition of the workers inhabiting the organization (Fritz & Lewis, 2025). It manifests through assorted features, entities, activities, and actions that institutionalize racial hierarchy whereby workers whose orientations deviate from White logics are relationally subordinated, limiting their agency and creativity (Bonilla-Silva & Lewis, 2025; Brown & Homan, 2024). This suggests that organizational processes that can reduce racial disparities must go beyond mere recruitment of individuals of racial groups to ways by which structural racism may operate to shape the cognitive, affective, and practice orientations of these racially diverse workforce (Kyere & Fukui, 2023; Ray et al., 2023).

Building on Ray’s (2019, p.27) conception of organizations as “constituting and constituted by racial processes that may shape both policies of the racial state and individual prejudice,” we argue that without intentional or conscious focus on race in research, diversity practices will not reveal how structural racism relies on race to structure organization to uphold whiteness. In the current paper, we specifically interviewed racialized minority mental health workers about their perception of organizational diversity. We seek to generate insight on ways organizational structural racism shape organizational dynamics among racially diverse workforce to strengthen occupational segregation by race. We also aim to identify potential pathways by which organizational processes can be designed to create supportive organizational culture to strengthen the agency and effectiveness of racially diverse mental health workforce.

In the following sections, we provide the context informing our study, followed by the theoretical framework—the theory of racialized organizations—guiding the study. Next, we discuss literature reviewing the racialized character of healthcare, including mental health organizations. We then present a case study that explored Black mental health workers’ experiences/perspectives, methodology, analysis, and findings, followed by implications for advancing structural equity within the mental health workforce.

## Context of the Study

The outbreak of the COVID-19 pandemic, which revealed the enduring violence of structural racism, along with simultaneous unabated killings of Black individuals and other persons of color, sparked one of the largest global movements protesting structural racism in 2020. In the US, several healthcare institutions, organizations, and professional associations (e.g., the American Medical Associations, the American Psychiatric Association, the National Association of Social Workers, etc.) reckoned with the role of structural racism in the (re)production of health disparities and their complicity in sustaining racial inequities that drive unequal health outcomes (Schouler-Ocak et al., 2021; Kyere et al., 2022). For example, the American Psychiatric Association (APA) president, Geller, resolved to address structural racism in the professions’ diversity efforts, in response to the civil unrest that followed the police killings of Black individuals, especially the death of George Floyd. Additionally, Geller’s resolution was to respond to the disproportionate impact of COVID-19 on racial minorities (Wills, 2021).

Although mental health organizations may diversify their workforce toward equitable and just mental health outcomes, the racialized contexts of the larger society and healthcare institutions in general constrain their efforts to uphold White racial framing in practice (Jones, et al., 1970; Sabshin et al., 1970; Shim & Vinson, 2020; Baima & Sude, 2020). This can severely undermine the effect of a racially diverse workforce, and entrench, not dismantle racial inequities. For example, Alegría and colleagues’ (2016) review of studies intended to address mental and behavioral health disparities found three common assumptions that must be addressed to advance equitable and just practice in mental health: (1) improving access to healthcare services alone, without addressing structural racism will not reduce or eliminate disparities; (2) incorporating preferences of minorities in service planning and implementation must be aligned with structural-level changes in order to achieve results and (3) structural racism consciousness must drive the conceptualization and design of evidence-based interventions toward the elimination or reduction of health-related disparities (Alegría et al., 2016; Shelton et al., 2021). Therefore, it is important to understand the larger racialized social systems that structure and direct mental health organizations. Accordingly, the current study takes a conscious focus on race—a race-conscious analytic lens which assumes racial undercurrent over racial neutrality (Kyere & Fukui, 2023)—to investigate the mechanisms by which structural racism may operate through community-based mental health organizations to constrain the potential gains associated with a racially diverse mental health workforce to persist racial disparities in mental health service access and outcomes. To do that, we apply the theory of racialized organizations (Ray, 2019, 2023).

## Theoretical Framework: *Theory of Racialized Organizations*

Grounded in structural racism consciousness, the theory of racialized organizations posits that the US is a racialized social system that operates through multifaceted and interconnected institutions and a hierarchy-based system that situates persons racially constructed as non-White at the bottom of the hierarchy while situating those racially classified as White and subscribe to White racial orientation at the top (Battalora, 2021; Brown & Homan, 2024; Mills, 2022). Organizations (e.g., hospitals, schools, the courts, the police, etc.) are viewed as mezzo-level actors and entities of the racialized states and are designed to (re)produce white supremacy ideology through racial hierarchical relations to animate inequities that negatively affect the effectiveness of racially subordinated workers, which in turn affect their lives outside of work (Brown & Homan, 2024; Ray, 2023; Wooten, 2019). Structural racism weaves inequality into the norms, rules, and modus operandi of organizations to normalize racial segregation as objective organizational processes, credentialling white racial logic at the advantageous point of the occupational hierarchy (Brown & Homan, 2024; Byron & Roscigno, 2019; Ray, 2019, 2023). Structural racism is evident in policies and practices of organizations that appear to be race neutral and confer disproportionate negative impacts on racially marginalized workers (Ray, 2019; Kyere & Fukui, 2023). There are some conceptual underpinnings of the theory that are important for our current study.

## Assumptions/Concepts

First, the theory of racialized organizations assumes that organizations are messo-level structures of the larger racial system and are designed to uphold white logic. Organizations are driven by racialized bureaucratic processes and practices through the organizational valuation and pursuit of the ideal workers (workers with certain characteristics such as accent and emotional expressions that credential whiteness) (Ray, 2019; Kyere & Fukui, 2023). Whiteness goes beyond an embodiment of assorted norms and behaviors of individuals labeled White. From an organizational standpoint, whiteness is a frame of institutional logic that influences what is deemed professional, legitimate, and an accepted standard across organizational settings (Fritz & Lewis, 2025; Ray, 2019). It is an aspirational and dynamic element that structures both informal and formal dimensions of organizations. Although individual agency is important, whiteness of organization shifts focus from individuals within organizations to structural mechanisms of organization that normalize white supremacy ideology regardless of the sector, the industry or the people operating within the organization (Jones et al., 1970). Even progressive organizations can embody and reproduce white logic that even individuals labeled White may fail to meet (Fritz & Lewis, 2025).

Second, this theory argues that through organizational structuring, racial hierarchy associates with cultural norms and rules to shape or define organizational climate and culture and subsequently assign social and material resources to those who embody whiteness (Brown & Homan, 2024; Ray, 2023). Third, given the process by which organizational resources can be accessed, racialized organizations promote or inhibit the agency or effectiveness of racial groups (Fritz & Lewis, 2025; Ray, 2019). Workers who do not embody whiteness can be restricted to the degree that their creativity and innovation conform or deviate from whiteness. In this way, workers who are racialized and marginalized as minorities may, in theory, be used to attract clients from similar racial backgrounds; in practice, however, these workers become a means of outsourcing (i.e., racial outsourcing) clients from racially marginalized communities to serve the capital and corporate interests of organizations, a process that can perpetuate disparities in service outcomes (Wingfield, 2019; Kyere & Fukui, 2023). Fourth, in efforts to ensure that organizations effectively serve marginalized and under-resourced populations, racialized minority workers undertake tasks beyond the scope of their positions, stepping into roles and responsibilities for which they are not compensated (Nelson & Johnson, 2024). This results in *racial task burdens* where racial minority workers take the burden of advocacy and justice related initiatives aimed at internal organizational change (Kyere & Fukui, 2023). In the context of political or public pressure, these workers are also called on toward *equity labor* where the racialized organization depends on them to design programs and initiatives that project the organization’s image as being progressive and equity focused.

Overall, from the perspective of the theory of racialized organizations, organizations within the racialized system may function to enforce the social contract to connect workers oriented toward whiteness (majority of whom tend to be labeled White) to organizational resources (e.g., authority, social prestige, and material rewards) (Harris, 1993; Mills, 2015; Ray, 2019) over racial minority workers. Organizational rules, processes, and routines are the mechanized path by which structural racism may work to link workers to material and social resources that produce and reproduce race-based occupational hierarchy like the antebellum plantation era, where the preservation of whiteness was prioritized (Harris, 1993; Ray, 2019). Consequently, whiteness may be regarded as the legitimate way to measure effectiveness, success, and for retention to the exclusion of the distinct cultural, social, and linguistic capital that racialized minorities bring to the workplace (Kyere & Fukui, 2023). Yet, structural racism is often decoupled from the way organizations, including mental health organizations, may discuss inequality in service delivery and outcomes, assuming that their structures and operational procedures are neutral (Byron & Roscigno, 2019; Ray, 2019).

Thus, drawing on the theory of racialized organizations, we argue that mental health organizations as subsystems of the racialized US landscape serve to activate and normalize cultural climate and practices that uphold white racial construct to persist white supremacy today (Baima & Sude, 2020; Feagin & Bennefield, 2014). In this regard, mental health workers (regardless of their racial group memberships) are thus participating in racialized affairs to uphold a socially constructed, hegemonic racial hierarchy in which racial minority workers are subordinate and treated as inferior, while workers labeled White and embodying whiteness are superordinate and deemed superior (Brown & Homan, 2024). The theory calls for a race-conscious analytic framework (intentional centering of race and especially from the perspective of racially marginalized workers) to the study of organizations.

## Literature Review

Research has shown that racism impacts racial minority mental health workers. For example, Dill and Duffy’s (2022) study showed how structural racism functions to situate Black women within health and mental health organizations at the lowest level of organizational hierarchy as direct care workers. In this position, Black women have higher proximity to potential psychological and physiological risks and experience the lowest wage and limited organizational support. In corroborating Dill and Duffy’s findings, Rivera-Núñez et al. (2022) study also suggests that racial minorities are segregated into the lower level of the healthcare workforce, where they have limited autonomy, and are exposed to high risks that compromise their own health while providing critical care. For instance, participants in Rivera-Núñez et al.’s (2022) study shared that during the COVID-19 pandemic, their job roles and responsibilities could be dissolved and reassigned to different roles such as cleaning COVID-19 vents, mask distribution, and temperature taking all of which increased their exposure to the pandemic. Yet, they received limited organizational support and benefits, including wages.

Prior research also showed that racial capitalism functions to structurally segregate racial minorities at the bottom of the occupational hierarchy, where they are disproportionately exposed to risks that compromise their own well-being (Schnake-Mahl et al., 2021). For example, Schnake-Mahl et al. (2021) found that White essential workers’ exposure to the risk of infectious diseases such as SARS-CoV-2 was lower compared to US-born Latino workers, whose exposure was also lower than foreign-born Latino workers. This suggests how structural racism, mediated by nativity, operates through organizational design to structure racialized healthcare workers, situating non-citizen racial minorities, followed by citizen racial minorities, at the bottom of the occupational ladder.

More recently, Pullen et al. (2023) surveyed community mental health workers to investigate racial differences in workplace networks and support and how these factors may be linked to perceived organizational support. They reported that, compared to Whites, Black mental health workers indicated having limited social network at work, and lower levels of managerial support. A particularly important finding from this study was that Black employees have limited connection with their supervisors. Relatedly, Breslow et al. (2023) results of logistic regression models of survey data generated from healthcare workers in New York suggested that structural racism within healthcare organizations influenced ethnic minority workers’ exposure to COVID-19-related stress. Their findings showed that compared to White workers, racially minoritized workers reported limited autonomy, inadequate access to personal and protective equipment, and redeployment in ways that were marginally significant. This shows that for racial minority mental health workers to contribute effectively to addressing or reducing racial disparities in mental health outcomes, the way structural racism operates to shape mental health care organizational structure, processes, and practices to create racial segregation within mental health organizations must be tackled.

According to Griffith et al. (2007), despite the mounting evidence showing the depth and breadth of health inequities, healthcare professionals are less likely to believe that the policies of their organizations shaping their own behaviors, and that of their peers contradict their professional oaths and principles aimed at health equity. Instead, professionals, including mental health workers, have been concerned with cultural competency as a pathway to addressing disparities in health outcomes. Consequently, most interventions addressing racial/ethnic health disparities have been in the form of individual-level training and education, such as cultural competency training, education sessions, and in-service training to increase knowledge of different racial-ethnic groups. The results of these efforts have shown that culturally driven intervention at the individual level has limited effects on racial health disparities (2007). Viewing racial health disparities through institutional racism, Griffith and colleagues (2007) outline three primary reasons for taking a system or structural approach to address health disparities: (1) health disparities are rooted in the history of racism including racialized medicine and healthcare delivery, (2) healthcare as a social institution is networked with other societal institutions that are nested within regimes of racialized inequities that disproportionately affects racial minorities, and (3) the complexity of the history of health inequities and the system suggests that interventions should also be as complex as the problem. The history of racialized healthcare suggests that healthcare disparities, including mental health, are more structural-level issues than individual-level behavioral concerns.

The embeddedness of racism in healthcare systems throughout the history of the US suggests that structural changes in organizational policies, practices, and processes are needed to address racial health disparities, including mental health disparities. According to Moore and Bell (2011), diversity discourse neutralizes and conceals whiteness through language and practices that treat institutions as inherently white and exoticize, criticize, and compartmentalize the cultural object of racial minorities as contributions to presumable neutral “us” (p.604). Discourse about and around diversity rooted in color-blindness decontextualizes racism and race from their social structural realities. However, it simultaneously bolsters the activation and application of racialized meanings that entrench white racial framing to diminish the efforts and creativity of racialized minority workers (Kyere & Fukui, 2023). For example, results of research that surveyed healthcare workers within academic hospitals related to predictors, perpetrators, and recipients of race-based discrimination show that, organizationally, racial minority healthcare employees (85%) deal with negative racialized experiences in their interaction with organizational policies, co-workers, supervisors, and patients (Hennein et al., 2021). These experiences were associated with limited opportunities for upward mobility and high job turnover. A review by Hennein et al. (2021) also revealed that race-based discrimination ranged from 22 to 71% among healthcare workers. Such experiences include micro invalidation whereby racial minority workers are misrecognized for their professional credentials, uneven application of salary and promotion policies, and excessive surveillance by security and supervisors. In previous work examining the impact of diversity training on discrimination of healthcare workers from ethnic minority backgrounds in the UK, King et al. (2012) found an association between ethnic/racial discrimination and declined worker motivation and, subsequently, turnover decisions. Similarly, African American employees have reported racism-related encounters during the hiring process, and in their employment tenure, they continued to be racially discriminated against on promotion, performance evaluation, salary, and termination policies (White et al., 2021).

Overall, the literature shows that while a racially diverse workforce holds promise for addressing racial disparities in mental health service usage, delivery, and outcomes, because structural racism structure organizations to credential whiteness, without a conscious efforts targeting structural racism and its mechanism, the potential positive effects of a racially diverse workforce cannot be realized (Jones et al., 1970; Kyere & Fukui, 2023; Bonilla-Silva & Lewis, 2025; Fritz & Lewis, 2025).

## A Case Study

The case study was part of a larger mixed-methods study to understand turnover in community mental health care organization (CMHCO). The organization was in an urban area and provided a variety of mental health services for adults and children, including case management, assertive community treatment, home-based and school-based services, supported employment, medication management, integrated treatment for co-occurring disorders, and individual and group therapy. Because of the diversity of services and the diverse client base of this CMHCO, the workforce is also diverse. However, in our prior review, where we developed a conceptual model, structural racism, through whiteness, was noted to potentially contribute to high turnover rates among racial minority employees within organizations with a demographically diverse workforce (Kyere & Fukui, 2023). In particular, the model suggested that without conscious attention to structural racism, the benefits linked to a diverse workforce may not translate into equity in service recipients’ outcomes. The case study thus applied a race-conscious framework to explore Black mental health workers in a racially diverse CMHCO’s perception related to diversity in relation to turnover of racial minority workers. Turnover was the interest of the overarching goal of the parent study but for this study, we focused on African American or Black employees, especially on organizational diversity and what might retain Black employees. We purposefully recruited Black employees who have worked for at least 7 years in the CMHCO. Study procedures and protocol were approved by the authors’ institutional review board and conducted in accordance with the ethical standards laid out in the 1964 Declaration of Helsinki and its later amendments.

## Procedure

By March 2022, 41 African American or Black employees (who had stayed at the organization at for least 7 years) were identified, and an email invitation was sent to them to participate in a study for understanding why people stay at the organization for a long time. Of the 41 Black workers, 24.4% (*N* = 10) agreed to participate. The demographic characteristics of these 10 workers were average age = 52.7(SD = 6.9); average tenure years at the agency = 13 (SD = 4.8); 2 males (20%)/8 females; 4 married (40%)/6 single; 2 part-time (20%)/8 full-time; 4 exempt (40%)/6 non-exempt; and 5 direct providers (50%). The rest (*N* = 31) of the invitees either did not respond to the email invitation (*n* = 29; 3 email attempts and 1 phone call) or declined (*n* = 2 no interest without any specific reasons). Interviews were conducted via Zoom by the first author. Informed consent was attached to the initial email for participants to review. The informed consent was reviewed with the participant, and verbal consent was provided before the interview began. Interviews followed a protocol that began with a “grand tour” question (Spradley, 2016) to establish a rapport and an entry point into participants’ experiences at the organization (e.g., tell me about your role and what you do). A series of focused open-ended questions was asked to explore participants’ perceptions of why they have stayed at the organization, why others have left, past or potential future turnover considerations, their thoughts about diversity, and why they think diversity matters. Because we were particularly interested in workforce diversity from the Black mental health workers perspective, the current case study focused on participants’ conversations around diversity-related issues: *What are your thoughts about employee diversity and inclusion at the organization? Have these ever affected your decision to stay at this organization, and as* *Black employee [at this organization], what are some of the things you would suggest to your employer that could influence you staying at your job for longer?* Interviews were recorded and saved to a university encrypted server, transcribed, and de-identified. Interviews lasted between 30 and 90 min. Participants received a $25 electronic gift card for their time.

## Data Analysis

The research team used Maietta et al.’s (2021) *Sort and Sift, Think and Shift* (SSTS) analytic approach to analyze the data. SSTS combines tenets of grounded theory, phenomenology, narrative research, and case study tradition. The SSTS facilitates an iterative data analysis process where the data serve as a guide to establish and apply a common coding structure for further analysis. First, participants’ conversations around diversity at the organization and ways that the organization can retain them as Black employees were carefully identified and transferred into PowerPoint slides for a deeper initial review by the first author. Next, the transcripts were shared with the research team, and we applied the six strategies to data analyses discussed by the SSTS approach: seeing the data (the dimension of the data), thinking of the data (in relation to diversity), organizing the data (structuring participants’ thoughts around diversity and ways organization can retain them as a Black employee), comparing data, saying the data (using voice on Microsoft to say and hear the data), and detailing the data, where synergies are established to draw emerging themes to construct a coherent narrative from the data (Maietta et al., 2021). The research team engaged in a constructivist and collective inquiry process to generate codes, which were then subsequently categorized into broader categories. The SSTS approach’s iterative process captured participants’ diverse voices that underscore their nuanced experiences around diversity in the organization.

## Findings

The findings are organized around core themes that convey participants’ thinking on *why workplace diversity matters* and their *perception of diversity in the organization.* The next theme focused on how they are impacted by and impact the organization based on how they, as Black employees, perceive diversity in the organization. Furthermore, we developed subthemes related to how the perceived organizational diversity operates to drive their efforts toward service delivery and access. In all, participants shared that diversity in the organization matters because it enhances mentorship (e.g., diverse leadership) and strengthens cultural concordance with clients (e.g., diverse providers). In relation to their perception of diversity in the organization, as Black employees, they shared that while the mid-level and direct service providers or lower-level workers are diverse, management is less diverse (e.g., institutionalized *whiteness* in management).

The next theme, the *impact* of the whiteness of the leadership, highlights the extent to which Black employees are motivated or able to be promoted to upper management positions, which facilitate autonomy to leverage organizational resources to advance equity in service provision. Two subthemes under the impact; racial *task burden and racial outsourcing,* revealed some of the mechanisms by which institutionalized whiteness may function to divert the equity and justice intentions of Black mental health employees at community mental health organizations toward the corporate and racial capitalist interest. *Racial task burden* is when racial minorities engage in and become overburdened with tasks (usually outside of their normal tasks associated with their roles) that should have been institutionalized within the organizational culture toward equity and justice. *Racial outsourcing* occurs in the context where the presence of racial minorities within organizations is encouraged to make organizations appealing to service recipients classified as racial minorities for organizations to realize their racial capital and corporate interest instead of serving the justice and equity concerns of these populations (Kyere & Fukui, 2023). Below, we present the themes emerging from the data. Because of the small sample size, we mask gender identities with s/he in their quotes.

## Why Diversity Matters?

Participants shared that a diverse workforce, especially a racially diverse workforce, is important in mental health organizations for several reasons. A common theme across their reasons for the racially diverse workforce within mental health organizations centered on mentorship, representation to foster a sense of psychological safety, and connection with diverse clients. Below are some of the quotes from participants expressing these rationales for workforce diversity.

Well, I think it’s just good to see people who look like you as role models and have them as mentors. We have a mentorship program. But I don’t know of anyone [Black employees] that’s in it (S222).

I think diversity plays a big role in the sense of even with the clients. Some clients don’t want to work with a Black person. Some clients don’t want to work with White person. Some clients don’t want to work with gays (S223).

We did have an African American [an executive leadership title]. [S/he] ended up leaving, but [s/he] started [a committee, that was meant to foster inclusion] … If there is a person of color in upper management, this is what the person can do (S221).

I know that our clientele is about 80-85% African American. And I think the staff is probably 65% Caucasian. If they’re not going to be fair on how they make their regulations and how they make their policies, then basically if anything else then it’s just wrong (S219).

## Perception of Workplace Diversity (Whiteness of the Leadership)

In responding to the prompt, *what are your thoughts on employee diversity?*, participants shared that although the organization served predominantly African American clientele and was racially diverse at the mid-level management, it is predominantly African American at the lower-level positions, while upper management was predominantly White. Their perception suggests that leadership is characterized by institutionalized whiteness. That is, norms and practices that credential a white racial cultural frame as the standard for upper management who make decisions regarding practice modalities and implementations. The following quotes are how participants described whiteness at this CMHCO.

It’s [diversity] a pretty sticky situation. Even with the employees…there’s more Caucasian people that are supervisors. We probably only have 2 team leaders that are Black team leaders, and the others are assistants. And it has changed within the last 5 years, I would say. I wonder about it, but at the same time, I don’t let that be the main source. This question has been there a long time…. You know, why is there so many White people that are supervisors? (S214).

So, [the organization] has been a place that is typical of a lot of work environments in the state. It’s predominantly White management, administration, and predominantly Black front line direct service workers…. I mean, racism is alive and well. And it has been very active at [the organization] just as it has anywhere else (S216).

They [the organization] do a very good job of frontline workers reflecting the populations that they serve. But they need to continue doing that in management. We still have a lot of White women. And while I really applaud the agency for promoting women to positions of power, I think that we need to continue looking for people of color (S222).

I think a lot of people are used to this structure in social service, being majority White, majority White women. And at the top it’s usually majority White men. But in our case, I think they think it’s diverse cause it’s White women. And that’s diverse for them. That’s not diverse for us (S221).

## The Impact of the Whiteness of Leadership

In a follow-up question about how the whiteness of the leadership influences them in the organization, including access to leadership positions, the conversations revealed the impact in various ways, including turnover decisions. An impact that was echoed by several of them has to do with the decreased motivation for leadership positions. Some felt that because of the resistant nature of institutionalized whiteness at upper management, it might be a stressful experience should they seek promotion.

There have been times where a person has considered… And some just don’t even consider it because sometimes you come up against a roadblock. There’s a roadblock because being a Black person and being exposed to the way all people live. So, a lot of times, a lot of people don’t apply for these positions… cause it’s like you are up against a brick wall. It’s just like you put me in a nursing home before my time (S214).

I think you have to put forth a lot of effort to deal with people who are biased. It’s just been easier, you know, to just stay put, and not have to deal with a lot of the racism and the politics. It’s easier just to stay put and deal with what I know rather than trying to promote and move all around and face all the challenges. What I’ve watched is Black employees leave [direct staff position], attempt to promote within the company, and end up leaving within a year (S216).

Further probe into what “stay put” means the participant shared that is how they minimize exposure to potential racial encounters at upper management because of the resistant nature of institutionalized whiteness in general, which another participant describes as a “roadblock.” The quote below provides clarification of what “stay put” means.

I think people could just learn how to deal with racism and work. We have to keep jobs; racism can’t stop you from making an income. So, you just learn to deal with it. Like how I’ve learned to deal with it, is to minimize my exposure to it. So, rather than work on a team and have to deal with people where I got to do a lot more work than them every day, I’ve just stayed in the [direct staff position] because the majority of the staff are Black, and a good number of the clients are also Black (S216).

Related to the impact of the resistant character of institutionalized whiteness on the Black employees at mental health organizations, another participant described the way that they would get together to plan ideas aimed at improving service and outcomes, but such ideas could be rejected by upper management.

So, we… come up with an idea, formulate a process or plan… We need to take this to upper management because … we can’t just implement something for the entire agency. We don’t have that type of autonomy. We need to have it approved by upper management. So, we’ll put time and effort into this procedure or process we came up with and … they’ll be like, no, we’re not doing it (S221).

## Racialized Task Burdens

With the institutionalization of *whiteness* in orienting leadership decisions around organizational hierarchy and culture, Black mental health workers at organizations may be burdened and overwhelmed by their workload and expectations. While this may seem normal and consistent with their roles as frontline care workers, a critical analysis shows how their racialized status, and roles are carefully woven into organizational culture in ways that mask the racial undercurrent. Below are examples of how participants described ways they are burdened racially.

It’s like, okay, you come on to do one task, but it’s like something has changed, and it’s like you get another task put on you, and people are really not willing to do something else that might need to be done because of what they had come in on. So, they feel like they might get more work, and feel like they are getting picked on or it wasn’t the role that they were supposed to be doing. (S220).

Nobody stays in the role of [direct staff position]. They may stay on the job. They’ll transfer. They’ll do other things. They don’t stay in that role because [direct staff position] is like the lowest on the totem pole. And you don’t have to have a college degree. You only have to have a high school diploma and lived experience. I had too much to do and to get this work done. And it seemed so overwhelming (S217).

Although a participant expressed that they were encouraged to take time off, they described that the population they work with is very challenging and could be overwhelming. The quote captures how they felt burdened because of her/his role and the associated job expectations.

You know, my caseload is like 40 clients that I have to deal with on a daily basis because they are always in need of something. It can be overwhelming (S223).

The racialized task burden was particularly striking in the experiences of a participant who had a [mid-level managerial position]. It shows that in the absence of proportional racial representation, racial minorities may disproportionately be burdened with too many roles and responsibilities. Their experiences probably give clues as to why some of the participants felt unmotivated to apply for a higher-level position. The quote below captures this reality.

I do support work for clinical staff, so that they have more time in the field to do direct support work. I do a lot of administrative work and agency work as well. I like to make sure there’s representation throughout the agency.... Like we do all these things to try to incorporate culture and diversity throughout the agency.…. We had to create a committee within the agency to make the rest of the staff feel safe and to make them feel seen and included. (S221).

## Racial Outsourcing

Related to racialized task burden is racial outsourcing. This means application of racial concordance where racial minority service providers’ demographic characteristics make organizations appealing to service recipients of similar background but for the purpose of advancing corporate and racial capital interests instead of equity and justice (Kyere & Fukui, 2023). The following quotes reveal this theme in practice from the perspective of participants.

Most of the people that we serve are African American. So, there is instant identification and ease in communication… they [African American clients] don’t have the perception of, you know, s/he [the African American worker] don’t know nothing about me. S/he can’t help me, you know. I can speak clinically to them, or I could speak to them in the language of the culture. And that is very helpful. It has helped to deescalate a lot of situations or to build rapport (S218).

As a [direct service provider], my role is to ignite help, ignite hope. Talk with clients regularly, sometimes one on one, sometimes in group sessions. I help the person that we’re serving to understand that they are not the trauma that they’ve been through, that they can reclaim their lives, transform their lives, and get their lives back. So, a [direct service provider] is somebody who uses their stories and lived experiences to ignite hope and to encourage the people that we work with (S219).

I’ve just stayed in the [direct care] position because the majority of the staff are Black… A good number of the clients are also Black (S216).

## Discussion

In context stratified by structural racial hierarchy, organizations are critical mechanisms by which structural racism works to entrench racialization to (re)produce racial segregation among workers and in turn disparities in service usage and outcomes (Ray, 2019; Kyere & Fukui, 2023). Scholars contend that whiteness—a frame of institutional logic that influences what is deemed professional, legitimate, and accepted standard across organizational settings—is a fundamental mechanism by which structural racism shape organizations including mental health organizations (Kyere & Fukui, 2023; Bonilla-Silva & Lewis, 2025; Fritz & Lewis, 2025). These scholars argue that rather than seeing organizations as race neutral, it is important that we are intentionally conscious about the racial character of organizations to assess the mechanism by which structural racism rely on racial hierarchy to structure organizational processes and practices beyond even diverse racial demographics of the workers within an organization.

The current study applied this understanding by intentionally asking Black mental health workers in a racially diverse CMHCO for their perceptions on the process around diversity in the organization and the implications for Black employees. While participants noted racially diverse mental health workforce as essential for addressing racial disparities in mental health care (Alden, 2022; Lara-Cinisomo et al., 2024), they revealed that structural racism, mechanized through whiteness, shapes the organization’s process. Our study’s themes are (1) workforce diversity matters, (2) whiteness of the leadership as the perceptions of organizational diversity, and (3) the impact of the whiteness of leadership in the occupational hierarchy. Two related subthemes were identified from the third theme: (3a) racial task burdens and (3b) racial outsourcing. The first theme revealed the relevance that our participants attach to workforce diversity. The remaining four themes highlighted the mechanisms through which structural racism may operate to drive the efforts and effects of a racially diverse workforce. This structural racism may prevent racially diverse teams from fully contributing to more equitable mental health outcomes.

Consistent with the theory of racialized organizations as mechanized through whiteness and with documented trends in healthcare organizations (Dill & Duffy, 2022; Rivera-Núñez et al., 2022), racial minority workers are overrepresented in direct care roles, linking them to lower wages and heightened occupational risks, including emotional and psychological stress (Wingfield, 2019). As revealed in the experiences of the Black mental health employees in the organization for this case study, racially diverse mental health workforce can help *deescalate situations or to build rapport, establish concordance with clients, and foster an organizational culture in support of workers belonging.* However, given that structural racism is highly prevalent and shapes organizational processes, our participants show that without an intentional focus on the mechanism by which structural racism operate within organizations, racial demographic diversity among workers within organization will only provide organization the necessary ingredients for structural racism to thrive.

In the case of the CMHCO that this study focused on, participants perceive whiteness of leadership, which deals with the application of white logic as the standard and the subsequent identification of individuals who are perceived to embody white logic (Bonilla-Silva & Lewis, 2025; Fritz & Lewis, 2025; Ray, 2023), as the critical path by which structural racism works. Consequently, in a situation where racially diverse workforce is present, it is structured in a way that appears *predominantly White in management and administration with predominantly Black frontline workers* where the workers at the management level tend to be predominantly *women labeled White*. While this knowledge validates the theory of racialized organization, it shows that whiteness of the leadership is a critical mechanism by which structural racism enables racial occupational segregation in organization. Through the standardization of whiteness at the management level, racial minority workers at the lower strata may have limited room to demonstrate creative autonomy that may drive just outcomes in the design and delivery of service, which in turn can address disparities observed among service recipients (Kyere & Fukui, 2023). Such situations also affect various outcomes among racial minority workers beyond the workplace, including health (Wingfield & Chavez, 2020), access to organizational resources (Wilson, 2019; Kyere & Fukui, 2023), political power, and life expectancy (Roberts & Olson, 2013).

The impact of this organizational process, where whiteness is credentialed as the standard, is that racial minority workers are relied on to outsource clients of similar background to achieve the corporate racial and capitalist interest of the organization instead of justice and equity. This finding supports previous study where Wingfield (2019) reveals how hospitals, clinics, and other institutions engage in *racial outsourcing*. This is a phenomenon where racial minority mental health workers are disproportionately assigned tasks involving racially diverse clients or communities, which may be systemic across healthcare organizations (Wingfield, 2019). This understanding suggests that diversity in the form of numerical representation of racial groups without a conscious attention to structural racism, such diversity efforts may persist and re-strengthen disparities in service outcomes among the recipients. Relatedly, the study participants revealed that because structural racism operates to elevate whiteness within the organization, racial minorities, especially those who are not oriented to the logic of whiteness, experience racialized task burdens regardless of their position within the organizational hierarchy. As shared by participants, due to being lower in the organizational hierarchy (e.g., direct care providers), they are tasked with addressing the complex needs of racial minorities, whose situation tend to be rooted in the very structural racism (Cénat, 2023). This can be culturally and emotionally overwhelming given that in this role, these workers have limited autonomy for creativity as well as limited access to critical organizational resources.

The current finding also reveals how racial minorities at the mid or even upper-level management may experience racial task burden. This racial task burden can be seen as *equity labor* or *diversity work* where racial minority workers are expected to foster an inclusive environment—work that is seldom recognized or compensated (Nelson & Johnson, 2024). The expectation that these workers should manage the racial dynamics within organizations reflects a broader societal trend where the burden of addressing racism is placed on racial minority workers (Byron & Roscigno, 2019; Helms, 2017). This added responsibility not only leads to burnout but also perpetuates the racial inequities that the organization ostensibly seeks to dismantle (Abramovitz & Zelnick, 2022). As shared by study participants [*There’s a roadblock... It’s just like you put me in a nursing home before my time*] and [*how I’ve learned to deal with it, is to minimize my exposure to it…stay put],* racial minority workers may deploy symbols or metaphors to express the nature of racism they face and coping and negotiation strategies to organizational level racism.

Current findings make an important contribution by revealing a unique path, *stay put* that adds to the complex mechanisms by which structural racism affects racial minority workers’ decisions for promotion to leadership at the management level. While these workers may not leave the organization, they may not want to take up opportunity for upward mobility, not because they do not want to, but because of the perceived consequences linked to potential racialized experience at the upper management. This is an important insight that reveals another indirect pathway by which structural racism shapes organizational segregation through the coping and negotiation strategies that racial minority workers may devise in response to the racialized context of the workplace. Although we know that racism affects the hiring and promotion of racial minority workers (Kyere & Fukui, 2023; White et al., 2021), this indirect pathway is underexplored. It may suggest that structural racism operates at varying degrees within organization and that racial minority workers decision to promote may be based on the degree to which they perceive necessary organizational context that support their effectiveness and overall well-being. While this understanding is consistent with the tenets of the theory of racialized organizations, it highlights another mechanized path that needs further exploration and can be targeted at the organizational setting when we are conscious of the racial character of organizations.

The critical arguments our study makes are that these disproportional burdens toward racial minority workers at the organizational level are rooted in a larger racialized system and mechanized through the routine ways organizations operate, which is whiteness. As participants shared, although their organization has demonstrated significant efforts in addressing racial disparities (e.g., creating diversity, equity, and inclusion committees, diversifying leadership at the mid-level), the perceived challenges appeared to be persistent (e.g., employees of racial minorities in direct service roles do not have time to attend committee). This suggests that diversity initiatives that attend to individual-level differences, experiences, values, and worldviews, without a focus on equity-based initiatives where institutionalized norms and processes are reconfigured, are more likely to entrenched racial equity labor, and racial outsourcing that burden and exploit racial minority workforce instead of real gains toward equity (Hamilton et al., 2023; Lerma et al., 2020). As observed by Lerma and colleagues (2020) within a university setting, although racial minority workers may be equity focused, their equity initiatives tend to be blocked and denied by the logic of whiteness that drives organizational processes. However, in the context of external or internal pressure that threatens organizational image, racial minority workers are used to safe organizational image. Organizational change often focuses on structural adjustments, such as altering administrative arrangements for strategy, financing, operations, or accountability (Coid & Davies, 2008). This frequently involves creating new units, department or positions in public healthcare while merging or eliminating existing ones (Mongelli et al., 2020). Reporting structures and accountability may also shift along with new financial systems or legal reforms (Jalivand et al., 2024). These changes often introduce new terminology, such as commissioning, fundholding, foundation trust, and clinical governance, to describe the evolving landscape (Storey & Holti, 2020). However, these forms of changes may do little to advance equity without racial consciousness embedded into them (Bussey, 2020).

## Implications for Structural Change

The findings of this study suggest that merely increasing racial diversity in the mental health workforce through numerical representation is insufficient to address racial disparities in mental health outcomes. Structural racism within mental health organizations constrains the ability of a racially diverse workforce to function effectively. As Alegría et al. (2016) and Griffith et al. (2007) mention, addressing mental health disparities requires systemic interventions that confront the root causes of institutional racism rather than relying on personal-level diversity initiatives. The current study supports the argument that without a deep understanding of the racialized social systems that shape mental health organizations, efforts to diversify the workforce will continue to fall short of achieving equitable outcomes. Moreover, addressing the impacts of racialized organizational hierarchies and task burdens will require fundamental shifts in how organizations view and engage with race (Ray, 2019). Mental health organizations must adopt frameworks that are explicitly anti-racist and race conscious, moving beyond tokenistic diversity efforts to challenge the entrenched White supremacist structures that govern the workforce (Ray, 2019; Wooten, 2019). This includes implementing policies that transgress whiteness and ensure equitable treatment and opportunities, integrating the creative, culturally innovative, and justice-oriented ideas that racial minority workers bring into organizational structures and practices. Additionally, organizations must recognize and compensate for the additional emotional and cultural labor disproportionately shouldered by racial minority workers while also redistributing the responsibility for creating inclusive environments to all workforce members, not just those from marginalized groups.

As concrete strategies, consistent with client-centered practice and organizational management in mental health practice (Rapp & Poertner, 1992), we advocate for a conception of mental health organization that shifts from an institutional and corporate model of organization characterized by a hierarchy-based structure to one based on relationship and role complementarity. In this practice, the unique and differentiated knowledge, and experiences that a diverse workforce brings to the organization are seen as constitutive to the functioning of the organization. Organizational structure and norms are developed in response to the changing needs of diverse clients through a coordinated approach that prioritizes dialogue and reciprocal relationships within and between management and direct workers as well as clients. Within such an organizational structure, the diverse direct workforce is not seen as a means of racial outsourcing, rather, they are seen as the frontline advocates for diverse client communities whose wisdom from practice needs to be embraced to inform strategic decisions toward effective services in an equitable manner.

Additionally, aligning with our mental health service system that has promoted efforts to diversify the frontline workforce to address diverse client populations’ needs in our community, it is essential to diversify middle and upper management workers to support the diverse frontline workforce in mental health organizations. Such efforts can facilitate mentoring and professional development opportunities for a diverse workforce. Unfortunately, while the importance might have been recognized, the current racialized systems have continued to present barriers. For instance, the Association of Social Work Boards (2022) report revealed the significant racial disparities in clinical licensure passing rates (i.e., being Black compared to White is about one-half as likely to pass the social work clinical exam on their first attempt). This means that racial minority employees may have limited opportunities to move up the occupational ladder, which requires advanced credentials. Accordingly, the efforts require larger system-level approaches to promote a diverse workforce.

Furthermore, rather than seeing organizations as neutral and meritocratic, it is essential that researchers and program evaluators apply race-conscious frameworks to enhance understanding of the racial character of organizations for what they are: a *long-standing social context to manage and prioritize whiteness* (Ray et al., 2023). This understanding shift focus on anti-racist work from individuals as racist or bad actors to applying organizational rules and resource allocation, which are more consequential (Ray et al., 2023). Race-conscious framework also helps the diverse workers, including individuals who identify as White inhabiting mental health organizations as implicated subjects (Rothberg, 2019) but differentially situated in the service of racialized intent over and above their individual and collective interest. The idea of implicated subjects is important for workers within organizations to collectively explore ways that structural racism operates through organizational hierarchy to exploit the various identities to persist racial inequalities so that they can collectively work on restructuring organizational practices and processes toward justice and equity.

## Limitations and Future Research

While this study provides valuable insights into how structural racism within mental health organizations shapes a racially diverse workforce, it is essential to note its limitations. The sample for the case study was drawn from an organization, and the small sample size may limit the transferability of findings to other employees or organizations. Additionally, our research focused on perceived diversity and not structural racism at the organization per se. Future research is needed to apply the race-conscious framework (intentional centering of race and structural racism in organization) to examine the structural characteristics of mental health organizations and how the differentiated and intersectional identities of the workers are relied upon to drive organizational processes and practices to (re)produce inequitable access to organizational resources with implications for disparities in service outcomes.

Despite the limitations, the current study highlights the urgent need for mental health organizations to adopt race-conscious analytic and intervention frameworks that explicitly address structural racism. These organizations can create environments where racially diverse workforces are not only recruited but retained and empowered to contribute to more equitable mental health outcomes at both local and the larger system levels. Diversity initiatives will likely remain performative without such structural changes that intentionally center structural racism and how it drives organization process including diversity. More importantly, future work that employs metaphors and symbolic meanings to deeply explore how racial minority workers experience and navigate structural racism at the organizational level is needed. In the current study, terms such as *a brick wall* and *stay put* are symbolically used to capture the nature of organizational racism they experience and the coping and strategic mechanisms by which racial minority workers navigate racialized organizational spaces where whiteness is credential as standard. Application of symbols and metaphors to engage racial minority workers on the racial character of organizations may enable us to identify and institutionally support practical ways racial minority workers can thrive without injuries within organizations.

## Declarations

### Disclaimer

The content is solely the responsibility of the authors and does not represent the official views of NIH

## References

[CR1] Abramovitz, M., & Zelnick, J. R. (2022). Structural racism, managerialism, and the future of the human services: Rewriting the rules. *Social Work,**67*(1), 8–16.

[CR2] Alden, A. M. (2022). *2020 Vision: How State Agencies Promote Racial Equity in Mental Health Care* (Doctoral dissertation, Northeastern University). https://www.proquest.com/docview/2670066266?pq-origsite=gscholar&fromopenview=true&sourcetype=Dissertations%20&%20Theses

[CR3] Alegría, M., Alvarez, K., Ishikawa, R. Z., DiMarzio, K., & McPeck, S. (2016). Removing obstacles to eliminating racial and ethnic disparities in behavioral health care. *Health Affairs,**35*(6), 991–999. 10.1377/hlthaff.2016.0029 27269014 10.1377/hlthaff.2016.0029 PMC5027758

[CR4] Association of Social Work Boards. (2022). 2022 ASWB exam pass rate analysis. *Final report*.

[CR7] Bailey, Z. D., Feldman, J. M., & Bassett, M. T. (2021). How structural racism works—Racist policies as a root cause of US racial health inequities. *New England Journal of Medicine,**384*(8), 768–773. 10.1056/NEJMms2025396 33326717 10.1056/NEJMms2025396 PMC11393777

[CR8] Baima, T., & Sude, M. E. (2020). What White mental health professionals need to understand about whiteness: A Delphi study. *Journal of Marital and Family Therapy,**46*(1), 62–80. 10.1111/jmft.12385 31106870 10.1111/jmft.12385

[CR9] Battalora, J. (2021). *Birth of a white nation: The invention of white people and its relevance today*. Strategic Book Publishing.

[CR10] Bonilla-Silva, E., & Lewis, A. E. (2025). Mechanisms and mechanics of racial hierarchy: Focusing on the “How” of racism. *Ethnic and Racial Studies,**49*(2), 359–369.

[CR11] Braveman, P. A., Arkin, E., Proctor, D., Kauh, T., & Holm, N. (2022). Systemic and structural racism: Definitions, examples, health damages, and approaches to dismantling: Study examines definitions, examples, health damages, and dismantling systemic and structural racism. *Health Affairs,**41*(2), 171–178. 35130057 10.1377/hlthaff.2021.01394

[CR12] Breslow, A. S., Simkovic, S., Franz, P. J., Cavic, E., Liu, Q., Ramsey, N., Alpert, J. E., Cook, B. L., & Gabbay, V. (2023). Racial and ethnic disparities in COVID-19-related stressor exposure and adverse mental health outcomes among health care workers. *American Journal of Psychiatry,**180*(12), 896–905. 37941329 10.1176/appi.ajp.20220180 PMC11590748

[CR13] Brown, T. H., & Homan, P. (2024). Structural racism and health stratification: Connecting theory to measurement. *Journal of Health and Social Behavior,**65*(1), 141–160. 38308499 10.1177/00221465231222924 PMC11110275

[CR14] Bussey, S. R. (2020). Imperialism through virtuous helping: Baldwin’s innocence and implications for clinical social work practice. *Journal of Progressive Human Services,**31*(3), 192–209.

[CR15] Byron, R. A., & Roscigno, V. J. (2019). Bureaucracy, discrimination, and the racialized character of organizational life. In *Race, organizations, and the organizing process* (pp. 151–169). Emerald Publishing Limited. https://www.emerald.com/insight/content/doi/10.1108/S0733-558X20190000060009/full/html

[CR16] Castillo, E. G., Isom, J., DeBonis, K. L., Jordan, A., Braslow, J. T., & Rohrbaugh, R. (2020). Reconsidering systems-based practice: Advancing structural competency, health equity, and social responsibility in graduate medical education. *Academic Medicine,**95*(12), 1817–1822. 32590465 10.1097/ACM.0000000000003559 PMC8279228

[CR17] Cénat, J. M. (2023). Complex racial trauma: Evidence, theory, assessment, and treatment. *Perspectives on Psychological Science,**18*(3), 675–687. 36288462 10.1177/17456916221120428 PMC10186562

[CR18] Coid, D. R., & Davies, H. (2008). Structural change in health care: What is the attraction? *Journal of the Royal Society of Medicine,**101*(6), 278–281. 10.1258/jrsm.2008.080107 18515774 10.1258/jrsm.2008.080107 PMC2408629

[CR19] Damian, J. A., Armah, T., & Lee-Winn, E. A. (2021). An intersectional approach to understanding the mental Health challenges of America’s essential workers. Harvard Medical SchoolPrimary Care Review. http://info.primarycare.hms.harvard.edu/review/mental-health-essential-workers

[CR21] Dill, J., & Duffy, M. (2022). Structural racism and Black women’s employment in the US health care sector: Study examines structural racism and Black women’s employment in the US health care sector. *Health Affairs,**41*(2), 265–272. 10.1377/hlthaff.2021.01400 35130061 10.1377/hlthaff.2021.01400 PMC9281878

[CR22] Feagin, J. R. (2020). *The white racial frame: Centuries of racial framing and counter-framing*. Routledge

[CR23] Feagin, J., & Bennefield, Z. (2014). Systemic racism and US health care. *Social Science & Medicine,**103*, 7–14. 10.1016/j.socscimed.2013.09.006 24507906 10.1016/j.socscimed.2013.09.006

[CR24] Fritz, M., & Lewis, A. (2025). Whiteness and organizations: institutions, legitimacy, and hegemony. *Ethnic and Racial Studies*, 1–18.

[CR25] Gee, G. C., & Hicken, M. T. (2021). Structural racism: The rules and relations of inequity. *Ethnicity & Disease,**31*(Suppl 1), 293–300. 10.18865/ed.31.S1.293 34045831 10.18865/ed.31.S1.293 PMC8143846

[CR26] Glazer, G., Tobias, B., & Mentzel, T. (2018). Increasing healthcare workforce diversity: Urban universities as catalysts for change. *Journal of Professional Nursing,**34*(4), 239–244. 30055674 10.1016/j.profnurs.2017.11.009 PMC6069531

[CR27] Griffith, D. M., Mason, M., Yonas, M., Eng, E., Jeffries, V., Plihcik, S., & Parks, B. (2007). Dismantling institutional racism: Theory and action. *American Journal of Community Psychology,**39*(3–4), 381–392. 10.1007/s10464-007-9117-0 17404829 10.1007/s10464-007-9117-0

[CR28] Hamilton, L. T., Nielsen, K., & Lerma, V. (2023). Diversity is a corporate plan”: Racialized equity labor among university employees. *Ethnic and Racial Studies,**46*(6), 1204–1226.

[CR29] Harris, C. I. (1993). Whiteness as property. *Harvard Law Review*. 10.2307/1341787

[CR30] Helms, J. E. (2017). The challenge of making Whiteness visible: Reactions to four Whiteness articles. *The Counseling Psychologist,**45*(5), 717–726. 10.1177/0011000017718943

[CR31] Hennein, R., Tineo, P., Bonumwezi, J., Gorman, H., Nguemeni Tiako, M. J., & Lowe, S. R. (2021). They wanted to talk to a ‘real doctor’”: Predictors, perpetrators, and experiences of racial and ethnic discrimination among healthcare workers. *Journal of General Internal Medicine*. 10.1007/s11606-021-07143-3 10.1007/s11606-021-07143-3 PMC8475391 34561823

[CR32] Jalilvand, M. A., Raeisi, A. R., & Shaarbafchizadeh, N. (2024). Hospital governance accountability structure: A scoping review. *BMC Health Services Research*. 10.1186/s12913-023-10135-0 10.1186/s12913-023-10135-0 PMC10777527 38200541

[CR33] Jones, B. E., Lightfoot, O. B., Palmer, D., Wilkerson, R. G., & Williams, D. H. (1970). Problems of black psychiatric residents in white training institutes. *American Journal of Psychiatry,**127*(6), 798–803. 10.1176/ajp.127.6.798 5482873 10.1176/ajp.127.6.798

[CR34] Kim, R., (2022). Addressing the lack of diversity in the mental health field. National Alliance on Mental Illness. https://www.nami.org/Blogs/NAMI-Blog/March-2022/Addressing-the-Lack-of-Diversity-in-the-Mental-Health-Field.

[CR35] King, E. B., Dawson, J. F., Kravitz, D. A., & Gulick, L. M. (2012). A multilevel study of the relationships between diversity training, ethnic discrimination and satisfaction in organizations. *Journal of Organizational Behavior,**33*(1), 5–20. 10.1002/job.728

[CR5] Kyere, E., Boddie, S., & Lee, J. E. (2022). Visualizing structural competency: Moving beyond cultural competence/humility toward eliminating racism. *Journal of Ethnic & Cultural Diversity in Social Work*, *31*(3–5), 212–224. 10.1080/15313204.2022.2057379

[CR6] Kyere, E., & Fukui, S. (2023). Structural racism, workforce diversity, and mental health disparities: A critical review. *Journal of racial and ethnic health disparities*, *10*(4), 1985–1996. 10.1007/s40615-022-01380-w 10.1007/s40615-022-01380-w PMC9361976 35930174

[CR36] Lara-Cinisomo, S., Lopez, G. M., & Flores-Carter, K. (2024). Developing a culturally responsive mental health workforce for Spanish-speaking and Latina/Latinx birthing people with perinatal depression and anxiety. *an integrated approach to perinatal depression and anxiety in Spanish-Speaking and Latina Women* (pp. 207–222). Cham.

[CR37] Lerma, V., Hamilton, L. T., & Nielsen, K. (2020). Racialized equity labor, university appropriation and student resistance. *Social Problems,**67*(2), 286–303.

[CR38] Maietta, R., Mihas, P., Swartout, K., Petruzzelli, J., & Hamilton, A. B. (2021). Sort and sift, think and shift: let the data be your guide an applied approach to working with, learning from, and privileging qualitative data. *Qualitative Report*, *26*(6).

[CR39] Maume, D. J., Rubin, B. A., & Brody, C. J. (2014). Race, management citizenship behavior, and employees’ commitment and well-being. *American Behavioral Scientist,**58*(2), 309–330.

[CR40] McGuire, T. G., & Miranda, J. (2008). New evidence regarding racial and ethnic disparities in mental health: Policy implications. *Health Affairs,**27*(2), 393–403. 10.1377/hlthaff.27.2.393 18332495 10.1377/hlthaff.27.2.393 PMC3928067

[CR41] Mills, C. W. (2015). The racial contract revisited: Still unbroken after all these years. *Politics, Groups, and Identities,**3*(3), 541–557. 10.1080/21565503.2015.1053400

[CR42] Mills, C. W. (2022). *The racial contract (25th Edition)*. Cornell University Press.

[CR43] Mongelli, F., Georgakopoulos, P., & Pato, M. T. (2020). Challenges and opportunities to meet the mental health needs of underserved and disenfranchised populations in the United States. *Focus: Journal of Lifelong Learning in Psychiatry,**18*(1), 16–24. 10.1176/appi.focus.20190028 10.1176/appi.focus.20190028 PMC7011222 32047393

[CR44] Moore, W. L., & Bell, J. M. (2011). Maneuvers of whiteness: ‘Diversity’as a mechanism of retrenchment in the affirmative action discourse. *Critical Sociology,**37*(5), 597–613. 10.1177/0896920510380066

[CR45] Nelson, J. L., & Johnson, T. D. (2024). How white workers navigate racial difference in the workplace: Social-emotional processes and the role of workplace racial composition. *Work and Occupations,**51*(3), 362–407. 10.1177/07308884231176833

[CR46] Pullen, E., Fischer, M. W., Morse, G., Garabrant, J., Salyers, M. P., & Rollins, A. L. (2023). Racial disparities in the workplace: The impact of isolation on perceived organizational support and job satisfaction. *Psychiatric Rehabilitation Journal,**46*(1), 45. 36809015 10.1037/prj0000543

[CR47] Rapp, C. & Poertner (1992). Social administration: A client-centered approach. *Longman*.

[CR48] Ray, V. (2023). *On critical race theory: Why it matters & why you should care*. Random House Trade Paperbacks.

[CR49] Ray, V. (2019). A theory of racialized organizations. *American Sociological Review,**84*(1), 26–53. 10.1177/0003122418822335

[CR50] Ray, V., Herd, P., & Moynihan, D. (2023). Racialized burdens: Applying racialized organization theory to the administrative state. *Journal of Public Administration Research and Theory,**33*(1), 139–152.

[CR51] Rivera-Núñez, Z., Jimenez, M. E., Crabtree, B. F., Hill, D., Pellerano, M. B., Devance, D., Macenat, M., Lima, D., Gordon, M., Sullivan, B., Rosati, R. J., Ferrante, J. M., Barrett, E. S., Blaser, M. J., Jr., & Hudson, S. V. (2022). Experiences of Black and Latinx health care workers in support roles during the COVID-19 pandemic: A qualitative study. *PLoS ONE,**17*(1), e0262606. 10.1371/journal.pone.0262606 35041702 10.1371/journal.pone.0262606 PMC8765643

[CR52] Roberts, J. M., & Olson, R. (2013). *How economic freedom promotes better health care, education, and environmental quality*. Heritage Foundation Washington.

[CR53] Rothberg, M. (2019). *The implicated subject: Beyond victims and perpetrators*. Stanford University Press.

[CR54] Sabshin, M., Diesenhaus, H., & Wilkerson, R. (1970). Dimensions of institutional racism in psychiatry. *American Journal of Psychiatry,**127*(6), 787–793. 10.1176/ajp.127.6.787 5482872 10.1176/ajp.127.6.787

[CR55] Schnake-Mahl, A. S., Lazo, M., Dureja, K., Ehtesham, N., & Bilal, U. (2021). Racial and ethnic inequities in occupational exposure across and between US cities. *SSM-Population Health,**16*, 100959. 10.1016/j.ssmph.2021.100959 34805478 10.1016/j.ssmph.2021.100959 PMC8590507

[CR56] Schouler-Ocak, M., Bhugra, D., Kastrup, M. C., Dom, G., Heinz, A., Küey, L., & Gorwood, P. (2021). Racism and mental health and the role of mental health professionals. *European Psychiatry*. 10.1192/j.eurpsy.2021.2216 10.1192/j.eurpsy.2021.2216 PMC8278246 34134809

[CR57] Shelton, R. C., Adsul, P., & Oh, A. (2021). Recommendations for addressing structural racism in implementation science: A call to the field. *Ethnicity & Disease,**31*, 357. 10.18865/ed.31.S1.357 34045837 10.18865/ed.31.S1.357 PMC8143847

[CR59] Shim, R. S., & Vinson, S. Y. (Eds.). (2020). *Social (in) justice and Mental Health*. American Psychiatric Pub.

[CR60] Spradley, J. P. (2016). *The ethnographic interview*. Waveland Press.

[CR61] White, M. L., Henderson, D. F., Smith, S. G., & Bell, M. P. (2021). A new look at an old problem: A positive psychology lens on discrimination–identity builders and work-related outcomes. *Human Resource Management Review*. 10.1016/j.hrmr.2021.100858

[CR62] Wills, C. D. (2021). Addressing structural racism: An update from the APA. *Current Psychiatry,**20*(3), 43–46. 10.12788/cp.0110

[CR63] Wilson, W. J. (2019). When work disappears: New implications for race and urban poverty in the global economy. In *Celebrating 40 Years of Ethnic and Racial Studies* (pp. 220–240). Routledge.

[CR64] Wingfield, A. H. (2019). *Flatlining: Race, work, and health care in the new economy*. Univ of California Press.

[CR65] Wingfield, A. H., & Chavez, K. (2020). Getting in, getting hired, getting sideways looks at organizational hierarchy and perceptions of racial discrimination. *American Sociological Review,**85*(1), 31–57. 10.1177/0003122419894335

[CR66] Wooten, M. E. (2019). Race, organizations, and the organizing process. In *Race, organizations, and the organizing process* (Vol. 60, pp. 1–14). Emerald Publishing Limited. https://www.emerald.com/insight/content/doi/10.1108/S0733-558X20190000060001/full/html

